# Developing Universal Influenza Vaccines: Hitting the Nail, Not Just on the Head

**DOI:** 10.3390/vaccines3020239

**Published:** 2015-03-26

**Authors:** Lidewij C. M. Wiersma, Guus F. Rimmelzwaan, Rory D. de Vries

**Affiliations:** Department of Viroscience, Erasmus Medical Center, P.O. Box 2040, 3000 CA Rotterdam, The Netherlands; E-Mails: l.wiersma@erasmusmc.nl (L.C.M.W); r.d.devries@erasmusmc.nl (R.D.V.)

**Keywords:** influenza, universal vaccine, MVA

## Abstract

Influenza viruses have a huge impact on public health. Current influenza vaccines need to be updated annually and protect poorly against antigenic drift variants or novel emerging subtypes. Vaccination against influenza can be improved in two important ways, either by inducing more broadly protective immune responses or by decreasing the time of vaccine production, which is relevant especially during a pandemic outbreak. In this review, we outline the current efforts to develop so-called “universal influenza vaccines”, describing antigens that may induce broadly protective immunity and novel vaccine production platforms that facilitate timely availability of vaccines.

## 1. Introduction

Influenza viruses belong to the family of *Orthomyxoviridae* and are divided into three types: A, B and C. Influenza A viruses can infect many different species and are responsible for substantial morbidity and mortality during seasonal epidemics. Furthermore, a zoonotic spillover event of influenza A could potentially be the cause of a novel pandemic. Influenza A viruses are further divided into subtypes based on the surface glycoproteins hemagglutinin (HA) and neuraminidase (NA). According to the HA designation, viruses are classified into two groups. H1, H2, H5, H6, H8, H9, H11, H12, H13, H16, H17 and H18 are considered group 1 HAs and H3, H4, H7, H10, H14 and H15 belong to group 2 HAs. Influenza B, unlike influenza A, viruses have no animal reservoir and predominantly infect humans. Influenza B can be divided into two phylogenetic lineages: Yamagata-like and Victoria-like [1]. These viruses have lower mutation rates [2], but also contribute to seasonal influenza activity considerably. Influenza C viruses are rarely isolated and disease caused by these viruses is usually limited to mild symptoms in children [3,4].

Both seasonal and pandemic influenza viruses can have huge public health consequences. In 2003 in the United States alone the total annual economic burden of seasonal influenza was estimated to be $87.1 billion [5]. Introduction of novel subtypes of influenza A virus into the human population may lead to pandemic outbreaks, as has happened three times in the previous century: in 1918 (Spanish flu, caused by A(H1N1) viruses), in 1957 (Asian flu, caused by A(H2N2) viruses) and in 1968 (Hong Kong flu, caused by A(H3N2) viruses). The most recent influenza pandemic in 2009 was caused by an H1N1 influenza A virus of swine origin [6]. Each of these pandemic outbreaks of influenza was associated with excess morbidity and mortality. On several occasions, zoonotic transmission of avian influenza A viruses from birds to humans of subtypes H5N1 [7,8] H7N9 [9], H9N2 [10], H6N1 [11], H7N3 [12,13] and H10N8 [14] has occurred, sometimes leading to fatal disease. Although thus far human-to-human transmission remains limited for these subtypes, infections with H5N1 and H7N9 viruses in particular constitute a pandemic threat [15]. It has been demonstrated recently that only a limited number of mutations in the HA and the viral polymerases are required to make airborne transmission of highly pathogenic avian influenza A viruses of the H5N1 subtype possible [16,17,18]. Indeed, some of these adaptive mutations were already found in circulating H5N1 viruses [19]. Influenza viruses can acquire additional genetic changes rapidly, either by mutation or by reassortment with viruses adapted to replicate in mammalian hosts [20].

Currently, most existing influenza vaccines are produced using labor intensive and time-consuming production methods that rely on the availability of embryonated chicken eggs. In the face of an outbreak caused by a novel emerging subtype, these methods suffer from logistical problems that preclude an adequate response. The delayed availability of sufficient numbers of vaccine doses may have disastrous consequences for public health. As will be discussed in this review, the limitations of the current vaccines highlight the pressing need for game-changing vaccines that induce long-lasting immunity against a wide range of influenza viruses.

## 2. Current Vaccination Strategies: Hitting the Nail on the Head

Currently used vaccines to protect against seasonal and pandemic influenza virus infections predominantly aim at the induction of antibodies directed at specific sites on the highly variable head domain of the HA surface glycoprotein and to a lesser extent, the NA glycoprotein [21]. Since the error rate of influenza virus is high due to low fidelity of the RNA polymerase complex, mutations in the viral genome can accumulate quickly, under selective pressure, such as exerted by virus neutralizing antibodies induced by previous infections or vaccinations. Vaccines that induce HA globular head-specific antibodies will become less effective when mutations in HA accumulate to such an extent that vaccine-induced HA-specific antibodies can no longer recognize their target, a process known as antigenic drift. Currently used vaccines are generally trivalent; they contain components of two influenza A strains (H1N1 and H3N2) and one influenza B strain. The strains used in seasonal vaccines are selected annually approximately eight months before the start of the seasonal vaccination campaign. Selection of vaccine components is based on prediction of strains likely to circulate in the subsequent influenza season. Although some methods, such as mathematical modeling of influenza virus evolution [22], have been developed to aid this prediction, the recommendation of the best possible vaccine strains remains difficult [23]. When vaccine strains do not match the epidemic strains, this can potentially lead to higher morbidity and mortality [24].

Different formulations of inactivated vaccines are used for parenteral administration: whole virion, split virion and subunit vaccines. Split virion and subunit vaccines, initially developed to overcome adverse reactions associated with whole virion vaccines [25], were shown to be of comparable immunogenicity but were less reactogenic than whole virion preparations. Since the 1970s, inactivated trivalent split virion vaccines have most commonly been used as seasonal vaccines. Paradoxically, the efficacy of these vaccines in age groups that are most at risk (the young and the elderly) is actually lower than for healthy adults [26,27,28,29].

An alternative to the inactivated formulations are live attenuated influenza vaccines (LAIV) produced by reassortment of gene segments encoding the desired HA and NA glycoproteins and those of a cold adapted (attenuated) strain. As these vaccines are administered intranasally, they can induce mucosal immunity, in addition to systemic antibody and T cell responses, more closely mimicking the immune response induced after natural influenza virus infection. Because of low immunogenicity of inactivated vaccines in children, LAIV may be better suited to protect this age group [30]. Indeed it was shown that after intranasal administration of LAIV to children between 3–17 years of age, significant increases in B-cell and T-cell responses could be induced that were sustained at least one year after vaccination [31,32]. However, whether universal vaccination of all immunologically naive children is in fact an advisable strategy remains a topic of debate. Vaccination of children at risk of developing severe complications due to influenza is of course highly recommended, vaccination of otherwise healthy children not at risk for developing severe complications with inactivated vaccines may interfere with development of heterosubtypic immunity that is otherwise induced following natural infections [33,34].

Even though in some situations, such as childhood vaccination, LAIV may have advantages over trivalent inactivated vaccines (TIV) [35], they suffer from similar problems regarding production. Like inactivated vaccines, the production process of LAIV is still predominantly egg-based and difficult to scale-up. Vaccine-induced antibodies are also directed mostly against the head domain of HA and thus at risk of losing effectiveness in the face of antigenic drift or shift. In addition, LAIV production has some challenges of its own like the incompatibility of certain HA and NA combinations with the backbone virus to create a replication competent vaccine virus [36].

Clearly, currently available influenza virus vaccination strategies leave a lot to be desired. The induction of strain-specific antibodies directed to the head domain of the HA may be efficacious if the vaccine strains match the anticipated epidemic strains. In case of antigenic drift or in the case of a pandemic outbreak caused by newly emerging influenza viruses, the availability of vaccines that induce more broadly protective immunity is desirable to overcome this important public health issue. Such vaccines should induce antibodies against more conserved proteins or regions thereof and activate other arms of the immune system, like cell-mediated immune responses to conserved proteins. In other words, such vaccines should hit the nail (but not just on the head).

## 3. Novel Approaches

**Figure 1:**
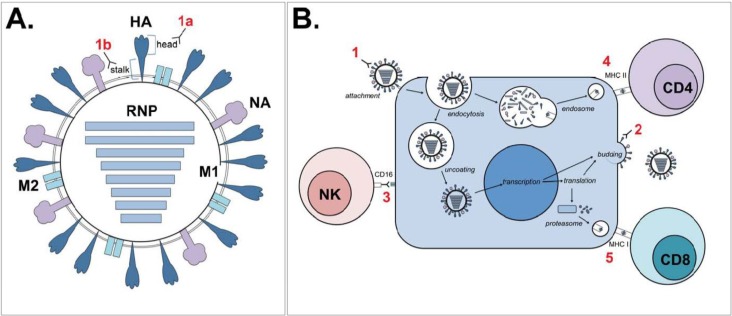
Schematic representation of possible immunological correlates of protection. Numerals in red show various immunological correlates of protection as indicated below. (**A**) Cartoon of an influenza virion, showing the hemagglutinin (HA) surface glycoprotein (stem and head), the neuraminidase (NA) surface glycoprotein, the matrix 2 (M2) ion channel, the matrix 1 (M1) structural protein and the ribonucleoproteins (RNPs: the combination of genomic RNA, viral polymerases PA, PB1 and PB2 and nucleoproteins (NP)). Antibodies directed against HA can either target the globular head (1A) or stem region (1B). (**B**) Interference of production of progeny virus by infected cells by various immunological correlates of protection, including (1) antibodies against HA head, interfering with binding or HA stem, potentially interfering with post-entry functions of HA, like endosomal membrane fusion; (2) antibodies against NA, limiting the production of progeny virus; (3) antibodies against M2e, HA or NA, followed by ADCC through CD16 signaling in NK cells (or phagocytosis, not shown); (4) virus-specific CD4^+^ T lymphocytes; and (5) virus-specific CD8^+^ T lymphocytes that possess cytolytic activity.

Compared to current regimens, novel approaches to vaccination against influenza can be improved in two important ways, either by inducing more broadly protective immune responses or by decreasing the time of production. To outline the current efforts in the field of development of so-called “universal influenza vaccines” in this review (Figure 1), we distinguish between two approaches: the approaches based on identifying targets that induce broadly protective immunity and those based on integrating these targets into novel vaccine platforms. Although these categories are artificial and there is of course substantial overlap between the two, together they roughly encompass the state of the art of universal vaccine development.

### 3.1. Approaches Based on Inducing Broadly Protective Immunity

The influenza genome encodes 11 proteins on eight segments of RNA: HA, NA, NP, M1, M2, NS1, NS2, PA, PB1, PB1-F2 and PB2. These proteins are variably conserved among avian and human virus isolates, as was shown in a large study that analyzed more than 36,000 sequences of virus isolates collected over the past 30 years. PB1-F2, NA, M2, NS1 and NS2 proteins were found to contain no sequences that were completely conserved in at least 80% of the viruses. However, comparison of the sequences of PB2, PB1, PA, NP, and M1 proteins showed that 55 sequences of 9–58 amino acids were completely conserved in at least 80%, or even as much as 95% to 100%, of the avian and human influenza A virus isolates and, although HA is generally considered to be highly variable, there was one 9 amino acid sequence that was conserved in all type A viruses [37]. This information may be of use when attempting to identify conserved targets required to make a universal vaccine, however simply broadening the response has already proved to be a challenge in itself, as further discussed below.

#### 3.1.1. Induction of Humoral Immunity

Antibodies can directly neutralize influenza viruses by binding to antigenic sites on the viral HA, therefore interfering with binding to the cellular receptor. As previously mentioned, most neutralizing antibodies induced following natural infection or vaccination target the highly variable globular head of the HA molecule. However, as a rule of thumb, these antibodies do not cross-protect against different HA subtypes or distantly related strains of the same subtype.

In addition to directly neutralizing virus particles, antibodies can also mediate a number of non-neutralizing immunologic functions (Figure 1B), including blocking of fusion between the viral and endosomal membrane, innate immune system activation, complement-mediated lysis, phagocytosis and antibody dependent cell-mediated cytotoxicity (ADCC). ADCC occurs when effector cells, in the case of influenza mostly natural killer (NK) cells, recognize the Fc portion of IgG bound to viral antigens presented on cell surfaces. The interaction of CD16 on NK cells with the Fc portion of IgG activates pathways that result in DNA damage and apoptosis, as well as release of granzyme B, perforin and other antiviral cytokines, eventually leading to the death of the influenza virus infected cell [38]. Because these non-neutralizing antibodies are often directed at more conserved regions, they have received considerable attention in the quest for universal vaccine development. The viral antigens considered to induce a humoral immune response, either neutralizing or non-neutralizing, are discussed below.

##### 3.1.1.1. Hemagglutinin

HA is the surface glycoprotein responsible for viral binding to and fusion with the host cell membrane. Since HA is abundantly present on the viral particle, it is attractive for the host immune system to target this molecule with antibodies. To date, 18 different subtypes have been shown to exist, of which H17 and H18 have only been detected in bats [39,40].

HA0 is the precursor molecule that is cleaved at a specific proteolytic cleavage site into HA1 and HA2 [41]. The HA head is formed by the central portion of HA1, and the HA stem is formed by the N- and C-terminal parts of HA1 and the full ectodomain of the HA2 [42]. The HA0 cleavage site has attracted some interest as a vaccine target as it is essential for viral replication and therefore highly conserved among both influenza A and B viruses. For influenza B viruses, consisting of only two antigenically distinct lineages, it was shown that a conjugate peptide vaccine targeting the HA cleavage site induced an antibody response that was able to protect mice from challenge with both influenza B lineages, serving as a universal influenza B vaccine. A similar strategy was attempted for influenza A and achieved a decrease in mortality and morbidity but did not fully protect vaccinated mice [43]. Decreased mortality and induction of heterosubtypic immunity could also be demonstrated in mice immunized with a H3N2 fusion peptide [44].

Targeting epitopes on the globular head, instead of the stem, can be advantageous since they are readily accessible to antibodies. Though rare, conserved regions in the HA globular head do exist. Recently, a conserved epitope was identified in H3N2 influenza virus using a broadly neutralizing human antibody named F005-126 [45]. Other antibodies have been shown to interact with the conserved regions in the otherwise hypervariable receptor binding site of the HA globular head. C05 for example, recognizes small conserved elements in the receptor-binding site and was found to neutralize strains from multiple influenza subtypes, including H1, H2 and H3 [46]. Another human monoclonal antibody, designated CH65, was found to insert into the receptor-binding pocket, effectively mimicking the sialic acid receptor. CH65 was shown to neutralize 30 out of 36 H1N1 strains tested, however no heterosubtypic immunity was demonstrated [47]. Furthermore, the potentially cross-reactive antibody S139/1 has been shown to neutralize multiple HA strains and subtypes, including H1, H2, H13 and H16 [48]. Further studies are obviously required, however these antibodies could provide clues for new targets in universal vaccine development.

Although inducing antibodies against the HA cleavage site or globular head may be of interest for universal vaccine development, by far most attention has focused on inducing antibodies against the stem region. Stem antibodies to group 1 influenza HAs can typically be induced by infection or vaccination, but remain subdominant to the head-specific antibodies due to limited accessibility of the stem [49,50]. Interestingly, infection of humans with pandemic H1N1, led to the induction of broadly neutralizing antibody responses against epitopes in the HA head and stem region, suggesting that induction of broadly reactive antibodies by a “universal” vaccine is a possibility [51]. Group 2 HA stem-specific antibodies have less frequently been reported in humans [52], although recently it was shown that 4% of H3 reactive human monoclonal antibodies from vaccinated individuals were able to cross-neutralize H7N9 viruses. These antibodies were shown to bind at least two sites on the stem region and were broadly reactive against group 2 viruses [53].

Alternative stem-specific antibodies that broadly neutralize group 1 influenza A viruses have been identified in humans and include CR6261 and F10 [54,55]. Similarly, antibodies CR8020 [56] and CR8043 [57] were hailed for their ability to broadly neutralize group 2 influenza A viruses. Broadly neutralizing antibodies effective against group 2 as well as group 1 HA molecules have also been recognized [58]. Based on the knowledge that stem-specific antibodies broadly neutralizing group 1 and 2 HA molecules exist in humans, it has been postulated that the design of a universal vaccine that induces a combination of broadly neutralizing stem-specific antibodies is possible and would effectively protect against infection with all influenza A viruses [56]. In addition to classical virus neutralization, these antibodies may also have alternative antiviral properties [59].

In order to induce antibodies that recognize conserved epitopes in the stem region various vaccination strategies have been employed, including the use of so-called “headless HAs”. These immunogens are essentially an HA molecule that lacks the globular head domain while maintaining the structural integrity of the stem. Mice were vaccinated with DNA encoding headless HA, followed by a boost with VLP containing headless HA protein and challenged with PR8. This headless HA vaccination strategy resulted in production of immune sera with broader reactivity than when full-length HA was used. Although sterile protection was not achieved, mice challenged with homologous virus showed no mortality and were partially protected against weight loss [60].

An alternative strategy to induce stem-specific antibodies involves repeated vaccination with chimeric HA molecules that contain the same stem region but that differ in their globular head domains. Using this strategy, the antibody response to the stem region is boosted with each vaccination while the response to the globular head is a primary response. These approaches show considerable promise as universal vaccine candidates [59,61,62,63,64].

##### 3.1.1.2. Neuraminidase

NA is a surface glycoprotein important in influenza virus release from cells. To date, 11 subtypes of NA have been identified, designated N1—N11. Since N10 and N11 have no specific NA activity, they are considered NA-like proteins [40,65,66]. NA appears to be more conserved than HA, however it is not immunodominant. NA-specific antibodies, commonly detected following natural infection or vaccination, are able to decrease severity of disease and reduce viral shedding. However, they are in general not capable of inducing sterile immunity [67,68,69] and NA-specific antibodies are not classically considered neutralizing as the likely mechanism through which they mediate the reduction of disease severity is by blocking virus release from infected cells [70]. Antibodies against conserved NA epitopes in the enzymatic active site have been shown to inhibit enzymatic activity of N1-9 as well as both influenza B lineages, but *in vivo* vaccination studies are still lacking [71,72]. The induction of NA-specific antibodies alone, regardless of how broadly reactive, is considered an undesirable strategy for universal vaccine production as it would result in an infection-permissive vaccine [73]. However, they remain of considerable interest as vaccine candidates in combination with other antigens. For example, it was shown that when administering a combination of multimeric forms of pandemic H1N1 HA and NA as a vaccine to ferrets, NA contributed very strongly to protection by HA [74]. Wohlbold *et al.* elaborate on the role of NA as a (universal) vaccine antigen in their review [75].

##### 3.1.1.3. Matrix 2 Protein

Matrix 2 (M2) protein is a relatively conserved transmembrane ion channel important for uncoating of the virus particle and has received significant attention as a candidate antigen for universal vaccine development. The extracellular domain of this protein (M2e) consists of only 23 amino acids and appears to be an attractive target due to its surface localization, high level of conservation and the linear nature of its peptide [76]. Since M2e-specific antibodies are thought to be mostly non-neutralizing, the proposed immunological mechanism that mediates the protection is ADCC, but also a role for alveolar macrophages has been demonstrated in a mouse model. Indeed the mode of action of M2-specific is dependent on the presence of FcγR [77]. Although much like NA, only negligible quantities of M2e-specific antibodies can be detected following natural infection, novel techniques appear to have overcome the initial immunogenicity issues [76]. The original Hepatitis B core protein-linked M2e vaccine candidate [78] was improved upon by expressing multiple M2e sequences in tandem [79]. This vaccine, in combination with different adjuvants, was immunogenic after intraperitoneal or intranasal administration and full protection against lethal strain X-47 influenza A challenge was shown in the mouse model. The combination of a production system that circumvents egg-based methods, the possibility of intranasal vaccination and the high immunogenicity has spurred phase 1 clinical trials with this vaccine (ACAM-FLU-A, Sanofi Pasteur) that have since shown both good safety and high levels of seroconversion [76]. Numerous other production systems have been tested for M2e, some of which will be discussed further under the novel antigen delivery heading.

##### 3.1.1.4. Internal Proteins

Non-neutralizing antibodies mounted against highly conserved internal proteins, such as NP [80,81,82], M1, PA, PB1 or PB2, may contribute to clearing influenza virus-infected cells, although the exact mechanism through which this clearance is mediated remains largely unclear. It was shown that NP may be transiently expressed on the cellular surface [83], thus offering a target for antibody binding and possible subsequent neutralization or ADCC. Further studies are required to elucidate the exact mechanisms of these interactions in the context of influenza virus infections or protection from infection. Although non-neutralizing antibodies to conserved proteins alone may not be sufficient for broad immunity, evidence is mounting that they are of importance in conjunction with, for example, cell mediated immunity [84,85]. Possibly, NP-specific antibodies lead to opsonization for improved antigen uptake by antigen presenting cells and subsequent antigen (cross) presentation although this could not be demonstrated *in vitro* [82].

#### 3.1.2. Cell Mediated Immunity

Although cell mediated immunity (CMI) to influenza virus infections does not prevent infection, it can significantly decrease viral shedding, reduce disease severity and mortality. As T lymphocytes (CD4^+^ or CD8^+^) tend to preferentially recognize the more conserved internal proteins, there is a greater potential for broad responses [86]. Indeed, CMI has repeatedly been shown to contribute to responses to both homologous and heterologous virus challenge [87,88,89,90]. Protection against virus of heterologous subtypes is known as heterosubtypic immunity [91], and it has been demonstrated that T cells play a crucial role in its development in animal models, such as mice [92], ferrets [93] and macaques [94,95]. In humans, a protective role of pre-existing virus specific CD8^+^ and CD4^+^ T cells has also been demonstrated against experimental infections [96,97] and natural infection with the pandemic virus of 2009 [98]. Studies employing adoptive transfer of T cells from primed donor to naive recipient mice have contributed to our understanding of the function of both CD4^+^ and CD8^+^ cells in protection [99]. A wealth of evidence exists for the importance of CD8^+^ cells in viral clearance. Conversely, CD4^+^ cells are thought to be of importance predominantly for the generation and maintenance of memory cells and for antibody production (reviewed in [100]).

Vaccines focusing on the induction of (CD8^+^) T cell responses have potential as universal vaccines [101], however there are a number of obstacles to overcome. To effectively induce an adequate CD8^+^ response, viral proteins must be endogenously produced in order to be efficiently processed and presented by major histocompatibility complex (MHC) I to CD8^+^ T cells. A number of novel vaccine platforms have been developed that allow for this, as will be discussed below. Another factor that should be taken into consideration when designing peptide antigen vaccines is the diversity of human leukocyte antigen (HLA) types as selected epitopes must be recognized by all HLA types to develop a truly universal vaccine.

The two most important internal proteins regarded as candidates for universal vaccine development are nucleoprotein (NP) and matrix 1 (M1) protein. The polymerase subunits PA, PB1 and PB2 have received less attention but may also be interesting targets.

##### 3.1.2.1. Nucleoprotein

The nucleoprotein (NP) is a structural protein that encapsidates the influenza virus RNA genome. It has key functions in RNA transcription, replication and packaging and is highly conserved among different influenza A subtypes. NP peptides presented on MHC class I molecules are among the most important targets for host CD8^+^ T cells [86,102]. Ways in which this knowledge is employed for universal vaccine development will be discussed further under the alternative vaccine platforms heading.

##### 3.1.2.2. Matrix 1 Protein

The matrix 1 (M1) protein is an influenza protein that plays important structural and functional roles in the viral life cycle. Structurally, it forms a layer between the viral envelope and the ribonucleoproteins (RNPs, the combination of genomic RNA, viral polymerase and NP proteins). Functionally, it drives viral budding and helps to control intracellular trafficking [103]. Highly conserved epitopes and consensus sequences have been employed in various vaccine platforms. An example of one such epitope of interest is the immunodominant HLA-A*0201 restricted M1_58-66_ epitope [104], which was recently shown to be presented not only by HLA-A but also by HLA-C molecules [105]. Of interest, this highly conserved epitope is under functional constraints, since mutations were not tolerated without loss of viral fitness [106].

### 3.2. Approaches Based on Novel Antigen Delivery Platforms

With progressively improved knowledge of the protective antigenic targets described above and the advent of new techniques, an ever-increasing number of platforms for the generation of a universal vaccine are becoming available. These techniques can be employed in numerous ways to deliver the desired antigens. Additionally, these systems often allow for different routes of administration, which in turn can steer the type of immune response induced. Novel platforms offer promise for both of the two improvements of influenza vaccines; induction of more broadly protective immunity and reduced production time.

#### 3.2.1. Viral Vectors

Viral vectors are recombinant, non-influenza viruses engineered to express influenza viral proteins. There are several advantages of using (attenuated or replication deficient) viral vectors as vaccine platforms for universal vaccines. As previously mentioned, cellular immune responses appear to play a pivotal role in protection from influenza infection, especially when a broad (heterosubtypic) immunity is desired. Subunit proteins, and whole or split inactivated viruses, often induce antibody and CD4^+^ T-cell responses due to exclusive antigen presentation by MHC class II. The advantage of using viral vectors is that they drive *de novo* synthesis of proteins in infected cells and facilitate endogenous antigen processing and presentation by both MHC class I and II molecules, thus inducing the complete spectrum of cellular and humoral immune responses. Additionally, they may stimulate mucosal immunity (depending on the route of administration) and thus function much like the existing LAIV vaccines, but without many of the safety or production issues. Viral vectors may actually be able to act as their own adjuvants as the immune system can mount a response to both the protein of interest and the vector. Consequently, these vaccines even have the potential to be used as bivalent vaccines, inducing immunity against the vector itself or its origin and the transgenic influenza virus protein(s). Conversely, this may require that the host is immunologically naive for the vector, as pre-existing immunity to the vector could potentially interfere with induction of immunity against the foreign protein of interest. Other concerns with some of the vectors, for example the use Newcastle disease virus, include the possibility of recombination and reversion to virulence in chickens [107].

DNA viruses, such as adenovirus [108], herpesvirus [109], baculovirus [110] and poxvirus [111], have been used for the generation of recombinant influenza virus vaccines. To date, vectors expressing influenza virus HA genes are most commonly used but recently other antigens, such as NP and M1, have also been employed, either alone or in combination with HA. Notably, Price *et al* have shown that immunization with a mixture of adenoviruses expressing NP and M2 confers heterosubtypic immunity [112], and can even reduce transmission in a mouse model [113]. The simultaneous expression of surface and internal proteins has the potential to induce both humoral and cell mediated immunity, therefore making these platforms interesting candidates for the development of universal vaccines.

One particularly promising candidate for universal influenza vaccine production is the Modified Vaccinia Ankara (MVA) vector [114,115]. MVA is avian adapted and does not produce infectious progeny upon infection of most mammalian cells [116]. It does, however, express early, intermediate and abundant late gene products in these cells, therefore making it both safe and effective. Of importance, potentially pre-existing immunity to the MVA vector induced after vaccinia virus vaccination against small pox before 1975, or after repeated vaccination with the vector, does not interfere with immunogenicity [117]. Many different influenza virus proteins have been expressed in MVA vectors; including HA of pandemic H1N1, H7N9 and H5N1 [118,119,120,121,122], HA combined with NP [117] and NP combined with M1 [123]. Interestingly, it was shown that priming with an adenoviral vector with NP and M1 followed by an MVA-NP-M1 boost, provided better heterologous protection than using either recombinant vector alone [124]. Recombinant MVAs have been evaluated in clinical trials and were shown to be effective, safe and practical alternatives to current vaccination strategies [125,126]. Therefore MVA is considered as a promising alternative vaccine platform for universal influenza vaccine development.

Beside DNA viral vectors, RNA viral vectors have also been considered for expression of influenza virus proteins, including paramyxovirus, flavivirus, retrovirus, coronavirus, alphavirus, bunyavirus and rhabdovirus [127,128,129]. RNA virus vectors have received less attention than DNA vectors as novel antigen delivery systems for universal influenza vaccines but have some interesting properties. Replication of RNA viruses takes place predominantly in the cytoplasm of infected cells, and therefore, they do not require translocation to the nucleus. In theory, this would allow for quicker dissolution of the vector. Furthermore, most RNA viruses (with the exception of retroviruses) pose no risk of integration into the host genome. However, these vectors are also less stable and have a smaller genome, which consequently also has a smaller capacity for introduction of transgenes.

#### 3.2.2. DNA Vaccines

Vaccination with DNA plasmids encoding influenza viral antigens has been investigated as a potential universal influenza virus vaccination strategy. Some of the practical advantages include rapid production, flexible antigen exchange and safety for use with highly pathogenic viruses [130]. Because antigens are expressed *in situ*, both humoral and cellular responses can be induced [131,132], as has been repeatedly shown in animal models [133,134]. Clinical trials have so far been only moderately successful [135,136,137], since plasmid DNA was poorly immunogenic in humans. Potentially, this could be improved by alternative delivery systems or the use of adjuvants. Regarding safety of DNA vaccines, concerns have been raised about integration of constructs into the vaccine recipient's genome as well as the possibility of inducing tolerance.

Newer techniques, such as self-amplifying mRNA-based vaccines, also appear to show promise in pre-clinical studies [138,139].

#### 3.2.3. Virus-Like Particles and Virosomes

Virus-like particles (VLPs) are composed of *in vitro* generated, self-assembling viral components that can serve as a vehicle for influenza viral antigens of choice (most commonly HA, NA and M1, but theoretically feasible for any antigen) [140,141,142]. They share some of the features of actual virus particles, but the most important difference is that, although they are able to enter cells, they are not able to replicate as they lack the viral genome. The lack of replication in the recipient has consequences for the type and the magnitude of immune response induced. The advantages are mostly practical, as it is an efficient, easy to produce, safe platform that potentially allows for rapid production of vaccines in pandemic situations [143]. This was confirmed during the 2009 H1N1 pandemic when phase II clinical trials were conducted in 4563 healthy adults that showed that the vaccine was both safe and immunogenic [144].

Virosomes can be considered a subset of VLPs, and are essentially influenza virus envelopes that lack the genetic material of the original virus. They consist of a phospholipid bilayer with incorporated surface glycoproteins (HA and NA). This vaccine platform is shown to be safe, potentially more effective than existing vaccines at inducing protective antibody titers, and has been licensed in several European countries. Virosomes predominantly induce a strong antibody response but have also been shown to offer some superiority over conventional subunit vaccines in the induction of cellular immune responses. It has been postulated that they may achieve these cellular responses through HA-mediated virosome entry into cells, mimicking infection, and delivery of antigen into the cytoplasm from where it can be presented on MHC class I molecules [145]. Additionally, the immunogenicity of virosomes can be enhanced with the use of (lipophillic) adjuvants [146]. However, their scope as universal vaccines is still limited as they are produced from detergent-treated whole influenza virus and thus face similar vaccine production constraints as existing vaccines [128].

#### 3.2.4. Adjuvants

A common limitation of some of the aforementioned novel platforms, but also of existing influenza vaccines, remains limited immunogenicity. Adjuvants, such as aluminum salts, have been used to boost immunogenicity of human vaccines for decades. Alum, however, has had only limited effects on improving immunogenicity of influenza virus vaccines. Oil-in-water combinations such as MF59 and ASO3 have been shown to be much more effective and are now licensed and used in seasonal and (pre)pandemic vaccine preparations, respectively [137,147]. Besides solely increasing immunogenicity, adjuvants can play an important role in facilitating increased speed of vaccine availability, because less viral antigen is required per dose (dose-sparing) meaning that more doses can become available in short period of time. In addition, adjuvants may help achieve the other aim of universal influenza vaccines; increasing the breadth of the immune response. It has been noted that oil-in-water adjuvants raise the quantity of cross-reactive antibodies [148], however this may merely be a reflection of the overall increase in the humoral response that also increases previously unnoticed broadly reactive antibodies to a level above their detection thresholds.

Conventional inactivated influenza vaccines induce virus specific CD8^+^ T cell responses inefficiently. As indicated above, these cells are highly cross-reactive and contribute to heterosubtypic immunity. The use of specific adjuvants may help to improve these cell-mediated immune responses. For example, some saponin based adjuvants, like ISCOMS, can translocate antigen across the membrane of antigen presenting cells and thus promote the endogenous route of antigen processing and presentation [149]. Indeed the use of this type of adjuvants results in increased virus specific CD8^+^ T cells responses as was demonstrated in animal models and in clinical trials [150,151,152,153].

## 4. Conclusions

We have reviewed some of the developments in the quest for a universal influenza vaccine. A few of these novel approaches have now advanced to clinical trials, where others have been tested in animal models and show promise. However, it should be noted that vaccination strategies depend on, or at least are influenced by, pre-existing influenza-specific immunity [49,154,155,156]. In the normal situation, humans are vaccinated and/or infected multiple times throughout life, resulting in a complex infection history shaping an intricate immunological landscape. It is virtually impossible to adequately mimic this situation in animal models and these models may therefore represent the situation of the immunologically naive population, which of course would be relevant for the use universal influenza vaccines in the pediatric population.

Influenza viruses continue to be an important threat to public health and although much progress has been made on the way to universal vaccines, many new approaches that show theoretical promise still have to be verified in both animal models and humans. The most promising universal influenza vaccines candidates are likely those that induce both broad humoral and cell mediated responses. Until true universal vaccines become reality, attention must be paid to pandemic preparedness and thus to increasing the speed of vaccine production. Platforms such as MVA show much promise for decreasing response time in an outbreak situation, but as for a truly universal influenza vaccine, we haven't quite nailed it yet.
